# PhySIC_IST: cleaning source trees to infer more informative supertrees

**DOI:** 10.1186/1471-2105-9-413

**Published:** 2008-10-04

**Authors:** Celine Scornavacca, Vincent Berry, Vincent Lefort, Emmanuel JP Douzery, Vincent Ranwez

**Affiliations:** 1 Institut des Sciences de l'Evolution (ISEM, UMR 5554 CNRS), Université Montpellier II, Place E. Bataillon – CC 064 - 34095 Montpellier Cedex 5, France; 2 Laboratoire d'Informatique, de Robotique et de Microélectronique de Montpellier (LIRMM, UMR 5506, CNRS), Université Montpellier II 161, rue Ada, 34392 Montpellier Cedex 5, France

## Abstract

**Background:**

Supertree methods combine phylogenies with overlapping sets of taxa into a larger one. Topological conflicts frequently arise among source trees for methodological or biological reasons, such as long branch attraction, lateral gene transfers, gene duplication/loss or deep gene coalescence. When topological conflicts occur among source trees, *liberal *methods infer supertrees containing the most frequent alternative, while *veto *methods infer supertrees not contradicting any source tree, *i.e*. discard all conflicting resolutions. When the source trees host a significant number of topological conflicts or have a small taxon overlap, supertree methods of both kinds can propose poorly resolved, hence uninformative, supertrees.

**Results:**

To overcome this problem, we propose to infer non-plenary supertrees, *i.e*. supertrees that do not necessarily contain all the taxa present in the source trees, discarding those whose position greatly differs among source trees or for which insufficient information is provided. We detail a variant of the *PhySIC *veto method called *PhySIC_IST *that can infer non-plenary supertrees. *PhySIC_IST *aims at inferring supertrees that satisfy the same appealing theoretical properties as with *PhySIC*, while being as informative as possible under this constraint. The informativeness of a supertree is estimated using a variation of the CIC (Cladistic Information Content) criterion, that takes into account both the presence of multifurcations and the absence of some taxa. Additionally, we propose a statistical preprocessing step called STC (Source Trees Correction) to correct the source trees prior to the supertree inference. STC is a liberal step that removes the parts of each source tree that significantly conflict with other source trees. Combining STC with a veto method allows an explicit trade-off between veto and liberal approaches, tuned by a single parameter.

Performing large-scale simulations, we observe that STC+*PhySIC_IST *infers much more informative supertrees than *PhySIC*, while preserving low type I error compared to the well-known MRP method. Two biological case studies on animals confirm that the STC preprocess successfully detects anomalies in the source trees while STC+*PhySIC_IST *provides well-resolved supertrees agreeing with current knowledge in systematics.

**Conclusion:**

The paper introduces and tests two new methodologies, *PhySIC_IST *and STC, that demonstrate the interest in inferring non-plenary supertrees as well as preprocessing the source trees. An implementation of the methods is available at: .

## Background

A phylogeny, or phylogenetic tree, is a representation of the evolutionary relationships among species. A well-known problem in biological classification is to combine phylogenetic information to produce more inclusive phylogenies. One way is to use supertree methods, which combine overlapping source trees, inferred from primary data (*e.g*. amino acids, SINEs or morphological traits). Supertree methods are also useful, teamed with supermatrix methods, in a divide-and-conquer approach to reconstruct very large phylogenies: first, the set of data is divided into subsets that are analyzed individually, then the resulting phylogenies are combined to reconstruct the global phylogeny [1,2].

Supertree methods can be classified into two categories, depending on the way they deal with topological conflicts, *i.e*. different arrangements of the same taxa among source trees. *Liberal *methods resolve conflicts, asking source trees to vote and opting for the topological alternative that maximizes an optimization criterion [3-7]. The hope is that each taxon is erroneously placed in only few source trees and this erroneous information will be overcome by the large number of source trees where the taxon is correctly placed. The most widespread liberal method is Matrix Representation with Parsimony (MRP, [3]). Supertrees proposed by liberal methods are often highly resolved and accurate, though several authors have shown that this approach sometimes leads to propose supertrees containing clades that contradict all source trees [8-10]. In contrast, *veto *methods do not allow the resulting tree to contain clades that contradict source trees. Some examples of veto kind methods are semi-strict consensus [8], SMAST and SMCT [11,12], *PhySIC *[13] and extensions of the strict consensus (*e.g*. [14,15]).

A recent method, *PhySIC*, returns a supertree with appealing theoretical properties. First, since it is a veto method, it does not contain relationships contradicting the source trees (*non-contradiction property*, denoted by PC). In addition, it only infers relationships that are present in a source tree or collectively induced by several source trees (*induction property*, denoted by PI). The last property insures that the method does not make arbitrary inferences. These features provide an unambiguous phylogenetic framework that is well suited for taxonomic revisions as for other applications where the reliability of the supertree is crucial.

**Figure 1:**
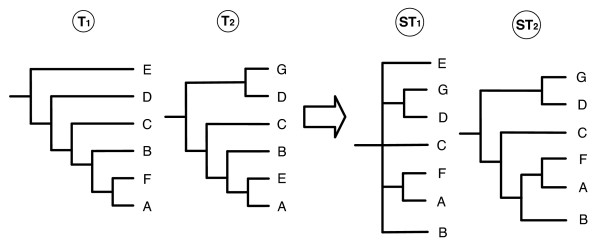
**In the case of trees displaying contradictions, such as *T*_1 _and *T*_2 _on the relative position of E, it can be preferable to propose a non-plenary supertree, such as *ST*_2_.** In this way, more information on the evolutionary relationships among the remaining species can be obtained. *ST*_1 _is inferred by MRP, *ST*_2 _by *PhySIC_IST*. *PhySIC *produces a star tree on this example.

**Figure 2:**
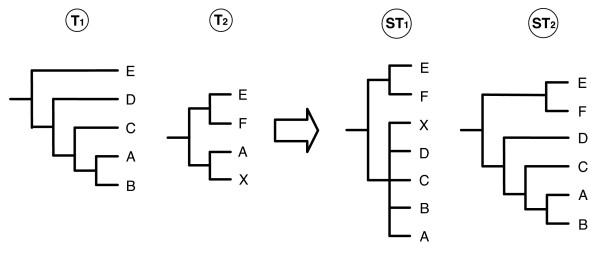
**In the case of trees displaying a significant lack of overlap, such as *T*_1 _and *T*_2_, it can be preferable to propose a non-plenary supertree, such as *ST*_2_.** In this way, more information on the evolutionary relationships among the species included in the supertree can be obtained. *ST*_1 _is inferred by MRP (the same tree is obtained by *PhySIC*), *ST*_2 _by *PhySIC_IST*.

Supertree methods, in particular veto methods, can propose unresolved supertrees, especially when the source trees do not sufficiently overlap and/or they present a high degree of contradictions (as gene trees affected by lateral gene transfers or tree-bulding artifacts, such as long branch attraction). When more informative supertrees are expected, a solution is to propose non-plenary supertrees, *i.e*. supertrees containing a subset of the taxa of the source trees. Figures 1 and 2 show two cases where proposing supertrees (*ST*_2_) lacking only one taxon provides more information on the phylogenetic relationships among other species. Both *SMAST *and *SMCT *methods [11,12] can produce non-plenary supertrees. The former consists in finding one of the largest taxa subsets *S *such that each input tree *T *proposes exactly the same resolution as the supertree for the taxa contained in *L*(*T*) ∩ *S*. In this approach the presence of a multifurcation in an input tree will inhibit resolution according to the information present in other input trees. On the contrary, the *SMCT *method allows these multifurcations to be resolved in the resulting supertree. Unfortunately, both underlying decision problems are NP-hard and no heuristic algorithm currently exists for general instances of these problems.

The algorithm presented in this paper, called *PhySIC_IST *(*PHYlogenetic Signal with Induction and non-Contradiction Inserting a Subset of Taxa*), looks for a supertree that satisfies PC and PI properties. *PhySIC_IST *allows multifurcations in input trees to be resolved thanks to the information present in other source trees. To deal with topological conflicts *PhySIC_IST *allows, like *SMAST *and *SMCT*, the insertion of only a subset of the species present in the source trees. Moreover, *PhySIC_IST *can also propose new multifurcations to avoid contradicting source trees, while *SMAST *and *SMCT *can only remove taxa. The aim of *PhySIC_IST *is not only to find *a *supertree *T *(plenary or not) that satisfies PC and PI but to find the most informative supertree satisfying both properties. Choosing the most informative alternative among several candidate supertrees requires one to be able to compare trees including potentially different subsets of the source taxa (such as *ST*_1 _and *ST*_2 _in figure 2). The informativeness of a candidate supertree is computed by a variation of the *CIC *(Cladistic Information Content) criterion [16]. This measure has roots in information theory and is basically proportional to the number of complete binary trees that are compatible with the evaluated supertree.

The resolution of supertrees computed by veto methods can be poor when considering large numbers of source trees. Indeed, adding more trees provides more information on the relative position of some taxa, but in the same time increases the number of local conflicts. To handle large collections of source trees, one has to resort to the liberal approach that allows to arbitrate between conflicts arising among source trees. The most common way to deal with incongruent source trees is to use a supertree method that takes ad-hoc decisions (according to a chosen objective criterion) in the face of individual conflicts met when building the supertree. The second and much less explored way is to preprocess the data according to a statistical procedure and then to apply a veto method, not contradicting the retained information that was estimated to be reliable. In this paper, we follow the latter approach that has the advantage of making the removing of conflicts between source trees explicit. More precisely, we introduce a preprocessing step to detect and correct anomalies in the source trees. This step, called STC (Source Trees Correction), analyzes the contradictions among the source trees; for all contradictions, it evaluates the possible topological alternatives and it drops the alternative(s) that is (are) statistically less supported (with a threshold chosen by the user). Then STC modifies each source tree (using a schema similar to that of *PhySIC_IST*–see *Methods*) so that it does not contain the dropped alternatives and yet remains as informative as possible. In other words STC aims at correcting the source trees that propose anomalous phylogenetic position for some taxa (due to lateral gene transfers, long branch attractions, paralogy ...). For example, if source trees contain two contradicting resolutions, one present in 99% of the trees and the other one present in 1% of the trees, we can reasonably think that the latter resolution is an anomaly and ignore it. If the user approves the proposed modifications, the *PhySIC_IST *veto method is then applied to the modified source trees. The resulting supertree satisfies both PI and PC properties for the collection of modified source trees. If the user is not satisfied with the modified source trees, he can change the threshold and restart the procedure, or choose to skip it. In this way, the liberal component of the supertree inference is not only made explicit but also interactive and parametrized. *PhySIC_IST *and STC were implemented using the *BIO++ *libraries [17], and are available from: .

## Results and Discussion

In this section we present results of large-scale simulations conducted to evaluate both the resolution and the accuracy of *PhySIC_IST *supertrees. These results help to measure both the improvement offered by *PhySIC_IST *on the previous version of the method, and the effectiveness of the *STC *preprocess. We also validate the new methodology by applying STC+*PhySIC_IST *to two biological case studies.

### Simulations

**Figure 3:**
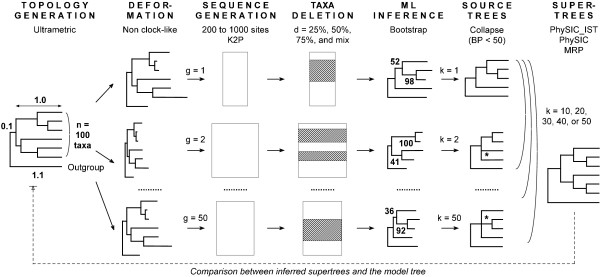
Simulation protocol.

The simulation protocol, depicted in figure 3, follows the standard guidelines in the field for assessing the effectiveness of supertree methods. Its details are inspired from [18]. We created 100 different clocklike trees; for each tree, every branch length was multiplied by a random value, chosen in an exponential distribution. Then each branch was divided by the total length of the resulting tree, providing 100 non-clocklike model trees. From each model tree, we generated 50 gene trees with different evolutionary rates, by multiplying every branch by a given value (the same within each gene tree, but different from gene to gene). Then the evolution of DNA sequences along these gene trees was simulated according to the K2P substitution model [19], obtaining a sequence alignment data set per tree. The different taxa overlaps observed in real data sets were simulated by randomly removing some sequences of those 50 data sets. As in [18,20], the deletion of sequences was performed according to four different proportions: *d *= 25%, to model a strong overlap between source trees, *d *= 50% and *d *= 75%, to represent sets with low taxon overlap, and a mixed deletion ratio (*d *= *mix*), to model a more realistic heterogeneity among source trees sizes. The mixed deletion condition is composed of one tenth of data sets with *d *= 25%, three tenths with *d *= 50% and six tenths with *d *= 75%. From the resulting data sets, we inferred 50 gene trees for each value of *d*, using PhyML [21]. The node supports were estimated using PhyML with a bootstrap process based on 100 replicates. For each inferred tree, we only retained the best supported nodes *i.e*. those showing a bootstrap proportion at least equal to 50. We built supertrees from all gene trees (*k *= 50) or only a subset of them (*k *= 10, 20, 30, 40). Combining this with the four deletion schemes, 100 data sets were obtained for each of the 20 conditions analyzed in this paper.

**Figure 4:**
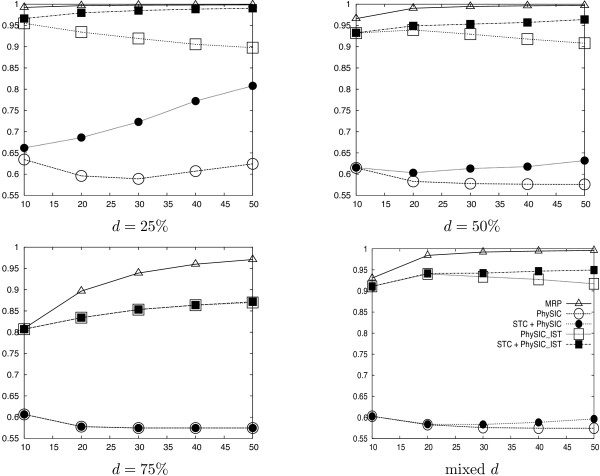
**Average *CIC*_*N *_values (y-axis) of supertrees built with different supertree methods (MRP Δ, *PhySIC *○, *PhySIC_IST *□, STC+*PhySIC *● and STC+*PhySIC_IST *▪), depending on the number of source trees (x-axis). **The results are shown for source trees inferred from data sets in which sequences have been deleted with *d *= 25%, 50%, 75% and mixed proportions.

**Figure 5:**
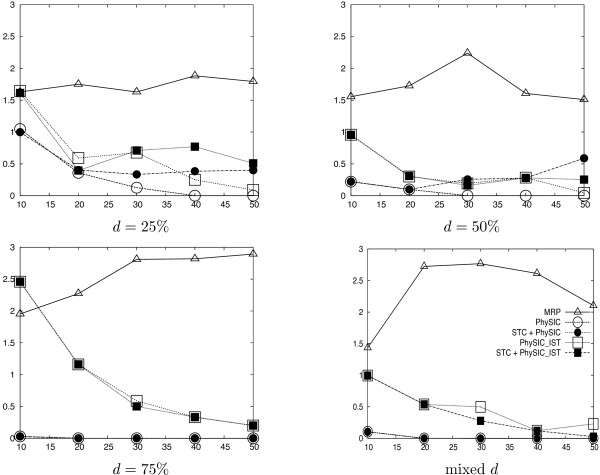
**Average percentage of type I error (y-axis) of supertrees built with different supertree methods (MRP Δ, *PhySIC *○, *PhySIC_IST *□, STC+*PhySIC *● and STC+*PhySIC_IST *▪), depending on the number of source trees (x-axis).** The results are shown for source trees inferred from data sets in which sequences have been deleted with *d *= 25%, 50%, 75% and mixed proportions.

We detail results for several supertree methods applied to the collections of source trees, namely *PhySIC *[13], *PhySIC_IST*, and MRP [3]. Veto and liberal methods are not really comparable because they are used for different purposes. Veto methods are expected to produce less resolved but more accurate supertrees: showing results for both kinds of methods gives an indication of how much is lost in resolution and of how much is gained in accuracy when using a veto method. For each supertree we evaluate its informativeness by computing its *CIC*_*N *_(see the *Methods *section for more details). Additionally, we compute its type I error, *i.e*. the number of triplets of the supertree not present in the model tree divided by the number of triplets in the model tree. For each condition, we average these values on the 100 replicates. Figures 4 and 5 summarize the results of the simulations.

The informativeness of supertrees is frequently compared using type II error, *i.e*. the number of triplets of the model tree that are not present in the supertree divided by the number of triplets in the model tree. It seems to us that the CIC_*N *_is more appropriate when comparing the informativeness of supertrees. Indeed, if a triplet *r *∈ R is included in the computation of the type II error, this may be a result of it not having been expressed in the supertree or of an alternative resolution having been proposed. To the contrary, the CIC_*N *_strictly measures the information contained in the supertree, whether it is accurate or not. The accuracy of the supertree is separately measured using the type I error. Because of this ambiguity of the type II error and for consistency with the optimization criterion of PhySIC_IST, CIC_*N *_graphics are provided instead of the type II error graphics.

#### Improvement of *PhySIC_IST *on *PhySIC*

The increase in resolution of *PhySIC_IST *in comparison to *PhySIC *is noteworthy (figure 4) no matter the deletion ratio. More precisely, the average *CIC*_*N *_of *PhySIC_IST *supertrees is 1.5 that of *PhySIC *(over all simulation conditions). Since *CIC*_*N *_is measured on a logarithmic scale, this means a considerable improvement on *PhySIC*. This different behaviour of the two methods is due, most of the time, to the fact that *PhySIC_IST *is allowed to infer non-plenary supertrees. Indeed, removing just one taxon is sometimes enough to make all source trees agree on a large subset of taxa. As veto methods are not allowed to contradict source trees, keeping the rogue taxa in the supertree means proposing a multifurcation for the surrounding subset of taxa, as done by *PhySIC*. The *PhySIC_IST *version escapes this situation by not including the rogue taxa in the supertree, and is hence able to obtain a relatively important resolution for the remaining taxa.

In the meantime, the type I error of *PhySIC_IST *(figure 5) is always inferior to 1% (except for *d *= 75% and *k *= 10) and decreases importantly as the number of source trees increases. From the experimental results, it could appear that there is a choice to be made between the two methods since PhySIC displays a significantly lower type I error rate (see figure 5), but this is mainly due to the fact that the trees reconstructed by *PhySIC *can be much less resolved, as expected from a plenary veto method applied to a large number of source trees. Thus, on practical data sets, *PhySIC_IST *is always to be preferred to *PhySIC*.

**Figure 6:**
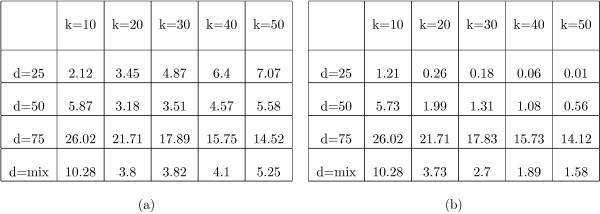
Average percentage of discarded taxa for supertrees built with *PhySIC_IST *(a) and STC+*PhySIC_IST *(b), depending on the deletion ratio and on the number of source trees.

The table in figure 6(a) shows the average percentage of source taxa not included in the supertrees inferred by *PhySIC_IST*, for each simulation condition. This percentage depends on the number and size of the source trees but remains globally low (*i.e*. less than 10%, except for *d *= 75% where it reaches ≈ 25%). When source trees contain insufficient information (*e.g*. *d *= 75% and *k *= 10), *PhySIC_IST *can infer supertrees lacking several taxa. Indeed, in such a case, the insertion of some taxa is impeded by the PI property: very little overlapping information is available and consequently many taxa cannot be placed unambiguously. Providing *PhySIC_IST *with more information (by increasing *k *or decreasing *d*) allows one to make the position of some taxa more precise, hence to propose larger supertrees. Yet, as the amount of available information continues to increase, the number of conflicts between source trees increases, leading some taxa no longer to be inserted due to the PC property. This means that increasing the amount of available information after some point can decrease the size of the inferred supertree (this phenomenon can be observed in the simulation results for *d *= 50% when increasing *k*).

The foreseeable but undesirable behavior of veto supertree methods when facing large numbers of source trees can be overcome by an explicit liberal preprocessing of the input trees, such as the STC proposed in this paper.

**Figure 7:**
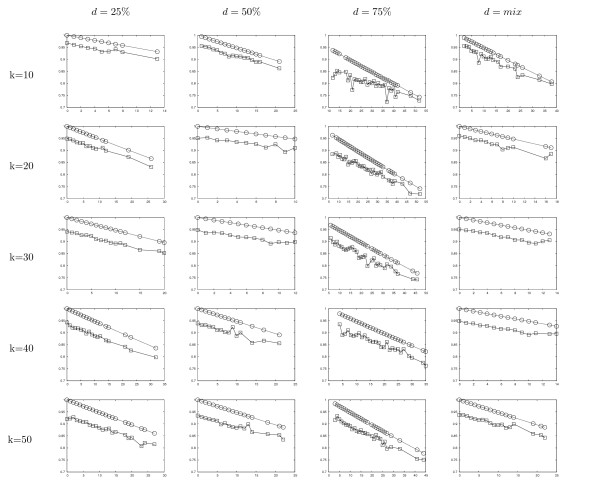
**Average CIC_*N *_values (denoted by □) plotted as a function of the number of input taxa not inserted in the supertree (x-axis). **Max CIC_*N *_values (denoted by ○) indicate the CIC_*N *_value of a fully-resolve tree with the same number of input taxa missing.

It is also interesting to analyze the CIC_*N *_values plotted as a function of the number of removed taxa. For each of the 20 conditions analyzed in this paper, the 100 inferred supertrees are split into classes, depending on the number of taxa not inserted in the supertrees but present in the source trees. Then, the average CIC_*N *_value is computed for each class (a class usually contains more than one tree) and these values are plotted as a function of the number of input taxa not inserted in the supertrees (see figure 7). For comparison, we also plotted the CIC_*N *_values of binary trees having the same number of leaves as the supertrees in each class. These values, denoted *max CIC*_*N*_, provide upper bounds for CIC_*N *_values of each class, hence enable to measure by eye the gap between *PhySIC_IST *supertrees and fully resolved supertrees of the same size. The plots obtained for the 20 tested conditions show the same trend with slight variations.

The CIC_*N *_values of the PhySIC_IST supertrees decrease as the number of "not-inserted" taxa increases, *i.e*. as the size of the supertrees decreases. This is expected given the role played by this number in the CIC_*N *_formula (see section *the CIC criterion*). More interestingly, *PhySIC_IST *supertrees overall have CIC values rather close to max CIC values, *i.e. PhySIC_IST *supertree are close to being fully resolved. Moreover, as the size of the supertrees decreases, CIC_*N *_values of *PhySIC_IST *supertrees and max CIC values decrease at a similar pace, the gap between both values narrowing slightly for the smallest supertrees. Thus, overall, the resolution degree of output supertrees appears to be only slightly dependent on the number of taxa inserted in the supertree. The only exception to this rule happens for the conditions *d *= 75 with *k *= 10 and *k *= 20. In these cases, which are the most extreme conditions in terms of overlap between the taxa set of source trees, the two curves decrease with different slopes. We now detail results obtained when resorting to STC statistical preprocess.

#### Efficiency of the STC preprocess

Figures 4 and 5 report simulations results for STC+*PhySIC *and STC+*PhySIC_IST*, when fixing the STC threshold to 95%, *i.e*. a 5% probability that a detected anomaly is not actually an anomaly (see the *Methods *section for more details). The resolution of both *PhySIC *and *PhySIC_IST *greatly increases thanks to the preprocessing step in most simulation conditions (25%, 50% and mixed deletion ratios *d*). The STC preprocess has no effect for *d *= 75%, where the low overlap between source trees impedes detecting anomalies.

STC+*PhySIC_IST *is on average 1.5 more informative than STC+*PhySIC *according to the *CIC*_*N *_measure. This replicates the gap observed between the methods without the preprocess, confirming the improvement of *PhySIC_IST *on *PhySIC*. The fact that the STC preprocess allows the *PhySIC *and *PhySIC_IST *supertrees to be more resolved without significantly changing the type I error, shows that this preprocessing step corrects the source trees in an appropriate way.

When only considering results with STC (Table (b) in figure 6), the average percentage of discarded taxa decreases with the number of source trees and increases when *d *augments. Thus, as more information is provided, supertrees are more and more informative, as usually happens with the liberal approach (*e.g*. see results for MRP in figure 4). Indeed, giving more information to STC brings out anomalies more and more clearly, thus tends to modify the source trees more and more accurately.

#### Comparison of liberal and veto methods

As expected, the resolution of supertrees obtained with MRP tends to increase with the number of source trees. In fact, MRP is a liberal method and adding trees supplies more information. Unexpectedly, its type I error does not decrease considerably when adding more trees to the analysis.

As already mentioned, the resolution of supertrees inferred by the two veto methods tends to decrease when including more trees (figure 4, 25%, 75% and mixed deletion rates *d*). In contrast, their type I error decreases importantly as the number of source trees increases. By applying the STC preprocess to *PhySIC *and *PhySIC_IST*, the two methods behave like liberal methods, *i.e*. the resolution of supertrees increases with the number of trees, as already explained except for *d *= 75%). This behavior is less apparent for *PhySIC*. Indeed, when faced with an insufficient number of triplets to satisfy the PI property, *PhySIC *can not benefit from the improvement with respect to PC achieved by the STC preprocess.

Note that in all conditions, MRP provides trees that are, on average, more resolved than other methods. Thus, MRP appears to be the most liberal supertree method among those investigated. This is not a surprise as, when two alternative resolutions conflict with one another, the MRP parsimony criterion favors that supported by the highest number of source trees, while the STC preprocess favors a resolution only when it is statistically more supported than the other (see Methods section for a precise description of STC). However, favoring more resolved supertrees also leads to more errors in trees. Indeed, the type I error of *PhySIC *and *PhySIC_IST*, with and without STC preprocess, is smaller than to that of MRP (except for the marginal condition *d *= 75% and *k *= 10).

The important question of whether less resolved but more correct supertrees should be preferred to the opposite alternative, can only be answered by knowing the subsequent use of the inferred supertree (see [13] for a list of cases where the former alternative is to be preferred.)

Plots of the type II error are not presented but they show the same relationships between the analyzed methods.

### Case study focused on placental mammals

**Figure 8:**
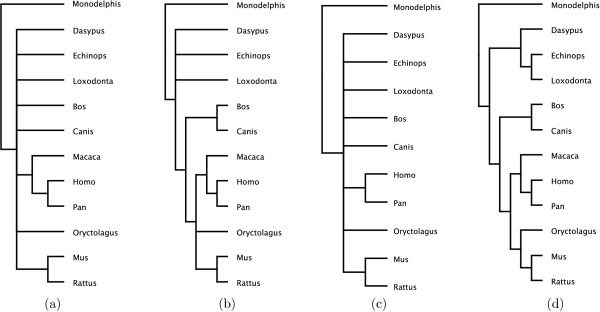
**Supertrees inferred by *PhySIC_IST *from two different collections of source trees.** Supertrees in (a-b) are produced by the *PhySIC_IST *analysis of 50 gene trees obtained from the OrthoMaM database queried for sequences longer than 3000 bp. Tree (a) is inferred without the STC preprocess while tree (b) is inferred with this preprocess, setting the threshold to 90%. Supertrees in (c-d) are produced from 157 gene trees inferred from sequences longer than 2000 bp. Tree (c) is inferred without the STC preprocess while tree (d) is inferred with STC, setting the statistical threshold to 90%.

To illustrate the effectiveness of *PhySIC_IST *and STC on biological data, we first considered data sets on 12 placental mammals. Primary data was obtained from the OrthoMaM database [22] that uses the EnsEMBL (release 41) orthology annotations to identify a set of exonic candidate markers for mammalian phylogenetics. The reliability of the phylogeny inferred from a single marker depends, among other things, on the length of the corresponding sequence alignment. Therefore, we only retained the DNA markers of OrthoMaM associated to the longest sequences, namely those having more than 2000 bp, which provided us with 159 sequence alignments. From the alignments, unrooted phylogenies were then separately inferred with PAUP* [23] using a maximum likelihood criterion. Using the facilities of our software, we rooted these trees according to one of the two following outgroups: *Monodelphis *or, if it was not present, *Dasypus*, *Echinops *and *Loxodonta *(see section *Methods *for more details). At this step, two of the 159 trees had to be discarded since they did not include monophyletic outgroups. A first supertree data set, called *ortho*2000, was composed of all these source trees. Additionally, we considered a second data set, called *ortho*3000, only composed of the trees obtained from alignment of more than 3000 bp. These two data sets respectively contain 157 and 50 trees, each tree including from 6 to 12 taxa. Figure 8 displays the supertrees inferred by *PhySIC_IST *on these data sets, with and without applying the STC preprocess. The STC threshold has been fixed to 90%.

With exons longer than 3000 bp, the *PhySIC_IST *supertree is extensively multifurcated, with only three obvious clades recovered (Figure 8(a)): the two muroid rodents (*Mus *+ *Rattus*), the two hominoids (*Homo *+ *Pan*), and the catarrhine primates (hominoids + *Macaca*). This reflects the fact that the source trees contain topological conflicts. A closer look at the source trees shows, for instance, that there is likely a long branch attraction phenomenon of the long muroid branch by the marsupial outgroup for the alignment composed of *Pan*, *Macaca*, *Mus*, *Rattus*, *Bos*, *Canis*, and *Monodelphis *exons orthologues to human exon 3 of the CELSR3-SLC26A6 gene (EnsEMBL transcript and exon references ENST00000383733, and ENSE00001498361). In the absence of the rabbit (*Oryctolagus*) orthologue that would break the muroid branch, *Mus *+ *Rattus *are artefactually attracted towards the basalmost position among placentals. This example illustrates the existence of conflicting resolutions among triplets of different source trees. Thus, without the STC preprocess, satisfying the PC condition results in a highly multifurcated supertree. In contrast, applying the STC preprocess leads to a more resolved supertree (Figure 8(b)). The two remaining multifurcations involve (i) the rabbit relative to muroids and primates, and (ii) the armadillo (*Dasypus*), elephant (*Loxodonta*), and tenrec (*Echinops*) relative to the other placentals. This probably reflects the lack of phylogenetic signal for these taxa among the 50 source trees.

With exons longer than 2000 bp, the *PhySIC_IST *supertree is extensively multifurcated, with only two obvious clades recovered (Figure 8(a)): *Mus *+ *Rattus *and *Homo *+ *Pan*. The greater number of source trees introduces additional conflicts within primates as compared to *ortho*_3000_. Additionally, the supertree lacks the taxon *Macaca*. The reason is that, in the source tree reconstructed from the ENSE00001300737 exon (EnsEMBL release 41), *Pan *is unexpectedly more closely related to *Macaca *than to *Homo*. This anomaly appears in only one of the 157 source trees, but this impedes pure veto methods from recovering the correct resolution for the clade. Indeed, inserting *Macaca *while preserving PC, implies losing the clade *Homo *+ *Pan*, hence leads to a completely multifurcated tree on the 12 taxa except for the trivial clade *Mus *+ *Rattus*. This supertree *T' *has a *CIC*_*N *_value inferior to that of the supertree *T *lacking *Macaca *(*CIC*_*N *_(*T*', 12) = 0.35 while *CIC*_*N *_(*T*, 12) = 0.435). For this reason, the taxon *Macaca *is not inserted. In contrast, STC+*PhySIC_IST *infers a plenary supertree (Figure 8(d)), the above-mentioned anomaly being overcome by a significant number of correct resolutions in other source trees. This supertree is also fully-resolved – unlike the supertree obtained from *ortho*_3000 _– as STC benefits from the signal of 107 source trees additionally present in *ortho*_2000_. The supertree topology is in agreement with the current view on placental phylogenetics which depicts the monophyly of euarchontoglires (rodents + lagomorphs + primates), laurasiatherians (*Bos *+ *Canis*), boreoeutherians (the grouping of the latter two clades), afrotherians (*Loxodonta *+ *Echinops*), and xenarthrans (*Dasypus*) + afrotherians [22,24-26].

### Case study focused on animals

The case study based on OrthoMaM only involved 12 species. To illustrate how PhySIC_IST performs on larger studies, we analyzed an animal phylogenomic data set containing 94 proteins (approximately 20,000 unambiguous amino acid positions) for 79 species, *i.e*. three poriferans (sponges), 5 cnidarians (sea anemones), and 71 bilaterians (chordates, urchins, mollusks, annelids, flatworms, roundworms, crustaceans, and insects) [27].

**Figure 9:**
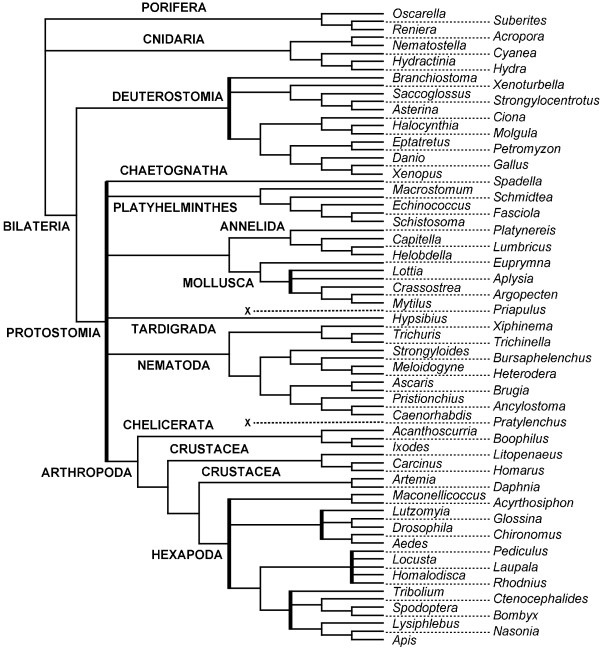
**Supertree reconstructed from the *PhySIC_IST *approach from 90 source trees of a phylogenomic animal data set.** The name of the major clades recovered are provided. The two species not incorporated in this non-plenary supertree are indicated by "X". Multifurcations are indicated by a thicker vertical line.

Individual maximum likelihood (ML) protein trees were inferred using Treefinder [28] under the WAG + Γ model of evolution. Among the 94 source trees, 4 (*rpl21*, *rpl37a*, *rpl38*, *rps17*) were discarded because the poriferan outgroup was not monophyletic. The remaining 90 ML topologies were subjected to a PhySIC_IST analysis. To choose the STC threshold, we varied the value of the threshold from 1 to 0.5 and we analyzed the CIC_*N *_values of the resulting supertrees. Fixing the threshold to a value from 0.84 to 0.69 leads to the most informative supertree. The topology of the obtained supertree (see figure 9) is in agreement with recent animal phylogenomic studies based on the ML and Bayesian concatenated analyses of conserved proteins under the WAG model of amino acid replacements [27,29]. For instance, bilaterians are split into protostomians and deuterostomians. Among protostomians, annelids group with molluscs, and crustaceans are paraphyletic due to the grouping of *Artemia *and *Daphnia *with hexapods. Among deuterostomians, Tunicata branches with Vertebrata, and *Xenoturbella *with Ambulacraria. Two taxa are not incorporated, the priapulid *Priapulus *and the nematode *Pratylenchus*. These two taxa are by far the less frequent and they are probably not inserted due to a lack of information. Seven multifurcations are displayed by the supertree. This reflects the fact that several source trees were inferred from very short alignments (*e.g. rps28a *possesses 54 sites). The resulting stochastic error yielded a lack of signal and/or contradictions on the position of some taxa, thus diminishing the supertree resolution degree. For instance, the multifurcation involving the 6 major protostomian lineages reflects the lack of strong signal under the WAG model, whereas the use of a mixture model like CAT provides increased topological resolution with monophylies of Lophotrochozoa (Platyhelminthes, Annelida, Mollusca) and Ecdysozoa (Tardigrada, Nematoda, Arthropoda) [27].

#### Conclusion

In this paper we propose a new supertree veto method (*PhySIC_IST*), running in polynomial time (see appendix in the supplementary material for details), that returns supertrees satisfying desirable theoretical properties (PC and PI). The simulations and the biological case studies confirm the practical effectiveness of *PhySIC_IST*, showing that this variant of *PhySIC *proposes supertrees that are much more informative than those inferred by the original *PhySIC *algorithm, while the type I error remains low (less than 1%). Additionally, we introduce a statistical preprocess of the source trees to detect and correct artifactual positions of taxa. This preprocess can be performed for any collection of source trees and hence benefits any veto supertree method. This approach has the advantage of separating the liberal resolution of conflicts among source trees from the assemblage of the supertree. This makes explicit the choices done to arbitrate between conflicting source trees, and allows the user to choose the extent with which the sources trees can be modified. In practice, STC+*PhySIC_IST *closes the gap between veto and liberal methods. This is the first practical method that provides informative and reliable non-plenary supertrees. The program is available for online executions and download at .

## Methods

### Definitions

We first recall notations used in the field, then we give a formal statement of the computational problem tackled by *PhySIC_IST*.

#### Notations

In this paper we only consider rooted phylogenies. This is not a limitation in general, as outgroups are usually available to root source trees prior to the supertree inference (see section *Rooting the source trees*). Given a tree T, we denote by *L*(*T*) the set of its taxa, each of them uniquely labeled. Given a collection T of trees, *L*(T) denotes the set of taxa appearing in at least one tree of T. A tree *T refines *a tree *T' *if and only if *T' *can be obtained from *T *by collapsing internal edges. Let *T *be a tree, and let *X *be a subset of its taxa. The subtree obtained from *T *by removing taxa not in *X *then deleting any vertex with only one child (except for the root of the tree) is called the *subtree induced by X *and denoted by *T*|*X*. For every three taxa we can have three different rooted trees, called *triplets*. We denote by *AB|C *the rooted tree that connects the pair of taxa (*A*, *B*) to *C *via the root. We say that a triplet *AB*|*C fits *a rooted tree *T *if *T*|{*A*, *B*, *C*} = *AB*|*C*. Any rooted tree can be decomposed into the set of triplets that fit it. We denote this set as *rt*(*T*). Thereby, *rt*(T) denotes the set of triplets that fit at least one tree of T, *i.e*.

$$rt(T)=\cup_{T_{i}\in T}rt(T_{i}).$$

A tree *T displays *a set R of triplets when R⊆rt(T); a set R of triplets is *compatible *if there is at least one tree *T *that displays R. A compatible set of triplets R induces a triplet *r*, denoted by R⊢r, if and only if all trees displaying R contain *r*.

#### The PI and PC properties

Given a collection R of trees and a tree *T *with *L*(*T*) ⊆ *L*(T), R(*T*, T) denotes the set of triplets of *T *for which *T *proposes a resolution; *i.e*. R(*T*, T) = {*AB|C *∈ *rt*(T) such that *rt*(*T*) contains at least one of the possible triplets on *A*, *B*, *C*}. We denote by $\bar{r}$ the triplets contradicting *r*, *i.e*. the two alternative triplets for the same set of three taxa present in *r*. If both *r *and at least one of the triplets contradicting *r *are present in *rt*(T), we say that the taxa of *r *are involved in a direct contradiction. Using these notations, we recall the PI and PC properties [13]:

• *T *satisfies PI for T if and only if for all *r *∈ *rt*(*T*), it holds that R(T,T)⊢r.

• *T *satisfies PC for T if and only if for all *r *∈ *rt*(*T*) and all $\bar{r}$, it holds that $R(T,T)⊢/\bar{r}$.

#### The *CIC *criterion

Since *PhySIC_IST *searches for the most informative supertree that satisfies PC and PI, it needs to estimate the information contained in a supertree *T*. For this purpose, we rely on a variant of the *CIC *criterion [16], related to the information theory. Let T be a collection of source trees on a ground set of *n *taxa. The information in an incomplete supertree *T *is a function of both the number *n*_*R*_(*T, n*) of its possible biological interpretations (*i.e*. the number of fully resolved trees on *L*(T) that encompasses *T*) and *n*_*R*_(*n*), the number of fully resolved trees on *n *leaves. More precisely, the *CIC *value of *T *relative to *n *source taxa is defined as:

$$CIC(T,n)=-\mathrm{lg}\frac{n_{R}(T,n)}{n_{R}(n)}$$

**Figure 10:**
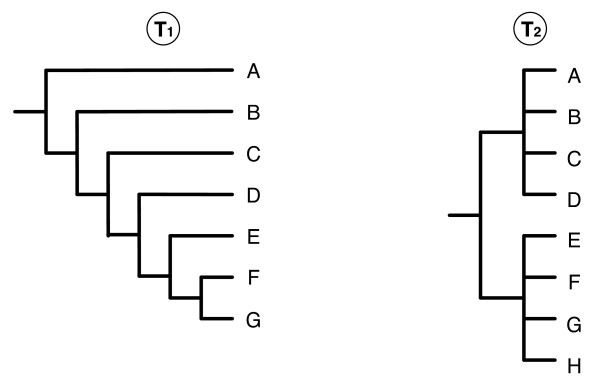
An example of different optimization criteria: number of triplets (|*rt*(*T*_1_)| = 35 while |*rt*(*T*_2_)| = 48) and the *CIC *criterion (*CIC*_*N *_(*T*_1_, 8) = 0.78 while *CIC*_*N *_(*T*_2_, 8) = 0.54).

In case of non-plenary supertrees, *n*_*R*_(*T, n*) depends on the multifurcations of *T *(since they reflect an ambiguity) and on the number of source taxa missing in *T *(since *T *contains no information for them). More formally, given a collection T of input trees and a candidate supertree *T*, the number of permitted binary trees for *T *referring to T is the number of binary trees *T' *such that *L*(*T'*) = *L*(T) and *T' |L*(*T*) refines *T*. We observe that, for each internal node *u*_*i *_with a number *c*_*i *_of children, we have (2*c*_*i *_- 3)!! possible resolutions [30]. Moreover, if *L*(*T*) ⊂ *L*(T), we have to insert all missing taxa, *i.e*. those in *L*(T) - *L*(*T*). A rooted binary tree of *i *taxa has 2(*i *- 1) branches; so, there are 2*i *- 1 possible positions for the (*i *+ 1)^*th *^taxon, taking into consideration the possibility of insertions above the root. We detail in the appendix how the value of *n*_*R*_(*T, n*) can be computed. In figures 4 and 10 we refer to *CIC*_*N *_(*T, n*) as the normalized value of *CIC*(*T, n*), *i.e*.

*CIC*_*N*_(*T*, *n*) = *CIC*(*T*, *n*)/(- lg1/*n*_*R*_(*n*)).

Another way to compare the information of different trees is to compare their number of triplets. However, the *CIC *criterion better takes into account missing taxa. For instance, consider the trees *T*_1 _and *T*_2 _in figure 10. The former is completely resolved but lacks taxon H, while the latter contains all taxa but is highly unresolved. Searching for the tree that maximizes the number of triplets, would lead to prefer *T*_2 _(since |*rt*(*T*_1_)| = 35 while |*rt*(*T*_2_)| = 48). However, it seems more reasonable to favor the tree that maximizes the value of the *CIC *criterion (in this case *T*_1_, since *CIC*_*N *_(*T*_1_, 8) = 0.78, while *CIC*_*N *_(*T*_2_, 8) = 0.54).

#### Statement of the computational problem considered

We previously explained why it is important that supertrees satisfy the PI and PC properties. Among the supertrees, that satisfy these properties, some may be more informative than others, as can be measured by the CIC criterion. This gives rise to the following optimisation problem:

**Problem **Most INFORMATIVE INDUCED AND NON-CONTRADICTING SUPERTREEE (MIICS)

**Input **a collection T of rooted trees.

**Output **a tree *T *such that:

(*i*)*L*(*T*) ⊆ *L*(T)

(*ii*)*T *satisfies PI and PC for T

(*iii*)CIC(*T*,|*L*(T)|) is maximum among the trees satisfying (*i*)-(*ii*).

We conjecture this problem to be intrinsically hard since it is a variant of the MIST (Maximum Identifying Subset of rooted Triplets) problem and of the ST (Triplet Supertree) problem, both shown to be NP-hard [31-34]. *PhySIC_IST *is a polynomial-time heuristics to solve the MIICS problem. Note that it is heuristics only on point (*iii*), since it always outputs a supertree satisfying (*i*) and (*ii*).

### Rooting the source trees

When *PhySIC_IST *is provided with unrooted source trees, it first has to root them. There are several approaches to root phylogenetic trees, among which are the outgroup, the molecular clock, and the non-reversible model of character-state changes. It has been shown that the outgroup criterion is consistently able to identify the root [35]. The software incorporates a rooting tool that automates the procedure. This tool accepts as input different levels θ_*i *_of outgroup, each one being a list of taxa. The rooting procedure considers each unrooted source tree separately. For a given source tree *T*, it determines the first θ_*i *_such that θ_*i *_∩ *L*(*T*) ≠ ∅. Then the tree is rooted on the branch leading to the smallest subtree hosting all outgroup taxa of θ_*i*_. If the proposed outgroup is not monophyletic, the tree *T *is discarded from the analysis. This procedure does not alter the resolution inside the ingroup nor in the different outgroup levels that can be present in the tree.

Rooting trees is not trivial, hence outgroup levels have to be chosen carefully.

### Inferring informative and reliable supertrees: PhySIC_IST

In this section we give the outline of the new method *PhySIC_IST*. This algorithm operates successive insertions of taxa on a backbone topology. Since it is a greedy algorithm, the order of the insertions has to be chosen carefully. Once a taxon is inserted, its presence in the supertree will never be questioned. It is therefore preferable to first insert the taxa with a strong and unambiguous signal. The first taxa inserted are thus those present in as many source trees as possible and involved in as few contradictions as possible. In fact, inserting a taxon that is present in numerous trees of T allows information, not only on its position, but also on the position of remaining taxa. On the other hand, delaying the insertion of incongruent taxa lessens the chances to misplace them due to incomplete information and to be unable to proceed with the insertion of remaining taxa. More formally, the priority order is determined as a function of R and $R_{dc}$, respectively the set of triplets of T and the subset of R that contains direct contradictions. Given a taxon *t*, we denote by |R(*t*)| (resp. |$R_{dc}$(*t*)|) the number of triplets containing *t *present in R (resp. $R_{dc}$). For each *t *∈ *L*(T) we compute the value

$$priority(t)=|R(t)|-|R_{dc}(t)|$$

and we order taxa in decreasing priority order.

Then, we build the starting backbone tree, formed of a root node to which are connected two leaves corresponding to the first two taxa in the priority list.

#### Supports

**Figure 11:**
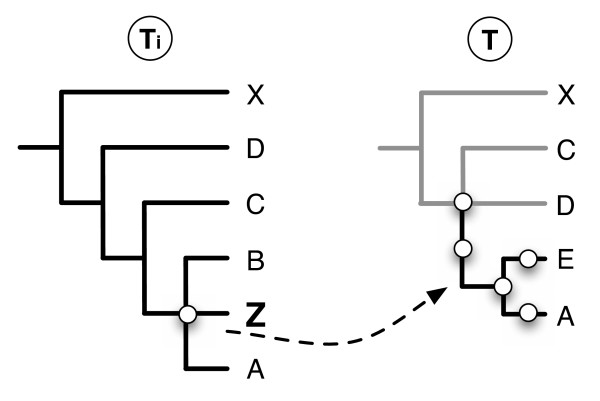
An example showing the supported region of *T *for the insertion of the taxon *Z*, according to tree *T*_*i*_.

Given a source tree *T*_*i*_, the backbone tree *T*, and a taxon *t *∈ *L*(*T*_*i*_) not yet inserted in *T*, we want to determine within which region of *T *the taxon *t *can be inserted without contradicting the information contained in *T*_*i*_. When the insertion of *t *on an edge (resp. a node) does not induce contradictions between *T *and *T*_*i*_, this edge (resp. node) is said to be *supported*. To delimit the supported region, we map the nodes of *T*_*i *_with the nodes of *T*. We define ${T^{\prime }}_{i}$ as *T*_*i*_|(*L*(*T*) ∪ {*t*}). We denote by ${f^{\prime }}_{i}$ the father of *t *in ${T^{\prime }}_{i}$ and by ${C^{\prime }}_{i}$ the set of children of ${f^{\prime }}_{i}$ other than *t*. The position of *t *in *T*_*i *_can be seen as delimited by ${f^{\prime }}_{i}$ as an upper bound and by each *c*_*i *_∈ ${C^{\prime }}_{i}$ as lower bounds. The corresponding bounds in *T *are denoted *f *and *C *(see algorithm 1 in the additional file 1 for more details and figure 11 for an example).

#### The different kinds of insertions

Once the algorithm has ordered the taxa in a priority list and built the seed backbone tree from the first two taxa, it proceeds with the insertion of remaining taxa in decreasing priority order. The easiest algorithm would be the one which chooses, at each step, the taxon whose insertion leads to the highest increase of the *CIC*, with the proviso that PC and PI remain satisfied. Unfortunately, this approach is too slow and unusable in practice. A faster way is to choose the best taxon, without testing all taxa, based on information already available. First of all, we are sure that, if all source trees support the insertion of a taxon in a region, inserting it in this region will not create contradictions between the source trees and the supertree. Thus this insertion will not violate PC. Additionally, if the region supported by source trees is not limited to a node or an edge, it means that the information we have is not enough to choose where the taxon has to be inserted. Such an insertion will surely violate PI. These considerations make insertions supported by all trees more appealing than insertions supported by only a part of them, and the insertions on a region well delimited more attractive than insertions on a larger region. This is the reason why in *PhySIC_IST *the insertions of taxa are done in four successive steps, each step being less restrictive than the previous ones in its requirements for inserting taxa. The strictest steps are done first, in order to maximize the chances for future taxa to be inserted and to maximize the *CIC *of the computed supertree. These four steps are differentiated according to two parameters, *all *and *cons*, each taking two values. The *all *parameter indicates whether taxa should be inserted only when a *maximum *support is observed for them somewhere in the backbone tree (*all *= *true*), or whether, in the absence of places with maximum support, places of *maximal *support should be considered (*all *= *false*). By maximum support at a position we mean that all source trees containing the taxa agree that it could be inserted at the given position. Note though that there might be several places of maximum support for inserting a taxon, due to a lack of overlap between the source trees and the taxa already in the backbone tree.

The case where *all *= *false *leads the backbone tree to temporarily contradict at least one source tree. This means that some of its edges have to be collapsed to ensure that the backbone tree still satisfies PC after the insertions. The collapsing of a minimal number of edges is performed by calling the *Check*_*PC *_procedure; an analogous test to check PI is performed calling the *Check*_*PI *_procedure [13]. If this collapsing decreases the value of *CIC *of the tree compared to its value prior to the insertion, then the insertion is cancelled. Overall, the insertions with *all *= *true *promise a more resolved supertree and are hence performed first, namely during the first two insertion stages, while the latter two run with *all *= *false*. The parameter *cons *indicates whether the insertion procedure should insert taxa only when there is a single best supported position for them (*cons *= *false*) or when *consensus *insertions are allowed (*cons *= *true*). A consensus insertion means inserting taxa on a node when all best supported places for the taxa are edges incident to the node. In this case, the insertion of the taxon *does not contradict *the source trees. Insertions with *cons *= *true *are always on a node, therefore insertions with *cons *= *false *are preferable because the possibility to insert taxa on a edge provides a tree with a higher *CIC *than an insertion on a node. Thus, for each value of *all*, a step with *cons *= *false *is first performed followed by a step with *cons *= *true*. During each insertion stage (see insertion procedure in the pseudo-code in appendix), all taxa not yet inserted in the backbone tree are considered. If the current taxon is inserted (by the roundIns procedure in the pseudo-code), then the algorithm tries to insert, always in priority order, all taxa previously considered that could not have been inserted before. These taxa have higher priority than taxa following the current one, and it is possible that the insertion of the current taxon enables the supported position for some of these taxa to be circumvented to a small enough part of the tree for their insertion to be possible. After each insertion the problematic branches are collapsed, to ensure that the backbone tree still satisfies PC. After inserting several taxa, the backbone tree may fail to satisfy PI. However, using the *Check*_*PI *_procedure to collapse problematic edges suffices to ensure that the backbone tree satisfies the property again. Collapsing branches with *Check*_*PI *_is done after each insertion stage and not after every insertion, contrarily to *Check*_*PC*_. The reason is that some edges of the backbone tree can fail to satisfy PI only temporarily and satisfy it again after the insertion of other taxa. On the contrary, if the backbone contradicts any source tree, it will keep contradicting it, no matter which taxon we insert afterward; it is thus preferable to detect this immediately to avoid problems that may arise while inserting remaining taxa. The improvement of *PhySIC_IST *on *PhySIC *shown in figure 4 is a consequence of three fundamental differences between *PhySIC *and *PhySIC_IST*. First, the new version operates successive insertions of taxa on a backbone and is not based on a revised version of the *Build *algorithm [36]; ergo, *PhySIC_IST *can frequently find relations between taxa that *PhySIC *cannot detect, being stopped in this analysis by a connected component of the Aho graph. In addition, the two methods do not have the same optimization criterion: indeed, *PhySIC *aims at finding the supertree satisfying PI and PC that proposes a resolution for as many triplets as possible, while *PhySIC_IST *looks for the supertree satisfying PC and PI that maximizes the value of *CIC*. Last, *PhySIC_IST *can propose non-plenary supertrees, *i.e *it will not insert the taxa that would decrease the *CIC *of the supertree, while *PhySIC *necessarily proposes a supertree that contains all taxa present in a least one source tree.

#### The STC preprocess

The aim of the STC (Source Tree Correction) preprocess is to analyze the direct contradictions in the source trees, to drop the statistically less supported alternatives and to correct the source trees accordingly. For a triplet *t*, we denote by $\dot{t}$ and $\ddot{t}$ the two other possible triplets for the same set of three taxa and by |*t*|, |$\dot{t}$| and |$\ddot{t}$| the number of occurrences of *t*, $\dot{t}$ and $\ddot{t}$ in the source trees. Only resolved triplets (like *AB*|*C*) are taken into account in the computation of |*t*|, |$\dot{t}$| and |$\ddot{t}$|, while star triplets are ignored. Given a set of source trees T, for each *t *∈ R(T), the vector composed by the three values |*t*|, |$\dot{t}$|and |$\ddot{t}$| is denoted by *occ*(*t*). We indicate with *max*(*t*) the maximum value in *occ*(*t*). Each time that *occ*(*t*) has at least two non-null coordinates, we have a direct contradiction. In this case, we want to drop the statistically less supported alternative(s), if any exists. To do that, the STC preprocess compares each non-zero value *i *in *occ*(*t*) with *max*(*t*) and it uses a Chi-Square test [37] with one degree of freedom to check whether the difference between the two values is significant. The null hypothesis **H**_0 _is that *p*_*i *_= *p*_*max*(*t*) _= $\frac{1}{2}$, *i.e*. there is no difference between the observed frequencies of the two triplets (one presents *i *times and the other *max*(*t*) times). For each *i*, the STC preprocess uses the basic Chi-square test to assess the plausibility of this hypothesis, computing

$$\begin{array}{c} \chi^{2}=\frac{{\left(i-np_{i}\right)}^{2}}{np_{i}}+\frac{{\left(max(t)-np_{max(t)}\right)}^{2}}{np_{max(t)}}\\ =\frac{{\left(i-\frac{n}{2}\right)}^{2}+{\left(max(t)-\frac{n}{2}\right)}^{2}}{2} \end{array}$$

where *n *= *i *+ *max*(*t*). This value is compared to the quantile corresponding to the threshold τ given by the user, *i.e. x*_0 _: *Prob*{*x *<*x*_0_} = (1 - τ). If χ^2 ^> *x*_0_, the STC preprocess rejects the **H**_0 _and inserts the triplet associated to *i *in (W(T)), *i.e*. the set of dropped triplets. Note that the two tests performed on each non-null coordinate are not independent. The user may use the threshold more as a setting parameter rather than interpret it as the probability that the STC drops a triplet that underlies a real anomaly. After that, the STC preprocess modifies the source trees applying *PhySIC_IST *to each *T*_*j *_∈ T, with R = R(*T*_*j*_) and R = (W(T)). In this way, we force the source trees not to contain the dropped triplets. Essentially, each modified tree may contain either new multifurcations, or lack some of its former taxa (if the phylogenetic position of these taxa changes extremely within the forest). Then *PhySIC_IST *is applied to the modified source trees. If the user does not agree with the source tree modifications, he can change *t *and restart the STC procedure or choose to skip it.

## Abbreviations

PC: Property of non-Contradiction; PI: Property of Induction; *PhySIC*: PHYlogenetic Signal with Induction and non-Contradiction; *PhySIC_IST*: PHYlogenetic Signal with Induction and non-Contradiction Inserting a Subset of Taxa; MRP : Matrix Representation with Parsimony; SMAST : Maximum Agreement SuperTree; SMCT : Maximum Compatible SuperTree

## Authors' contributions

VB, EJPD and VR initiated this research on non-plenary supertrees. CS designed the *PhySIC_IST *variant of *PhySIC *under the supervision of VB and VR. She conducted the simulations, whose results were analyzed by CS, VB and VR. EPJD and VR provided the data and analysis of the case studies. The idea of preprocessing the source trees was proposed by VR. The STC procedure was established by CS, supervised by Gilles Caraux.

VL supervised the creation of the *PhySIC_IST *web site. CS and VB participated in the creation of the *PhySIC_IST *web site.

CS, VB, VR and EPJD contributed to the manuscript.

## Supplementary Material

Additional File 1 **Outline of main *PhySIC_IST *subroutines and computation of the time complexity for *PhySIC_IST*.** Click here for file
