# Supplementary material for: PhySIC_IST: cleaning source trees to infer more informative supertrees
Source: BMC Bioinformatics. 2008 Oct 4;9:413. doi: 10.1186/1471-2105-9-413 (PMC2576265; doi:10.1186/1471-2105-9-413)
Supplement: Additional File 1 — Outline of main PhySIC_IST subroutines and computation of the time complexity for PhySIC_IST. [file 1471-2105-9-413-S1.pdf]

Appendix

Outline of main PhySIC_IST subroutines.

support(T_i, T, t)

$T_i^{'} \leftarrow T_i|(L(T) \cup \{t\})$;
$f_i^{'} \leftarrow$ the father of t in $T_i^{'}$;
$C_i^{'} \leftarrow$ the sons of $f_i^{'}$ (other than t) in $T_i^{'}$;
$I \leftarrow L(T_i^{'})$ - $L(subTree(f_i^{'}))$;
foreach $s \in C_i^{'}$ **do**
    $C \leftarrow$ C $\cup\ lca_T(subTree(s))$ // i.e. the lca in T of the taxa present in $subTree(s)$;
$f \leftarrow$ the lowest node in T s.t. $\forall s \in C$, $L(subTree(s)) \subseteq L(subTree(f))$ and $L(subTree(f)) \cap I \neq \emptyset$;
M $\leftarrow$ {m $\in$ children(f) s.t. $L(subTree(m)) \cap I = \emptyset$ };
suppOn(f) ++;
foreach $m \in M$ **do**
    foreach $u \in$ *subTree(m)* **do**
        if $\nexists s \in C$ *s.t.* $L(subTree(u)) \subset L(subTree(s))$ **then**
            suppAbv(u) ++;
            if *u is not a leaf* **then**
                suppOn(u) ++;

Algorithm 1: Procedure that increments the supports of edges and nodes of T, within the region where the taxon t can be inserted without contradicting the tree T_i.

Check$_{\text{PC}}$ $(T, \mathcal{R}, \mathcal{R}_{dc})$

$\mathcal{R}_t \leftarrow rt(T)$;
foreach $r_T \in \mathcal{R}_T$ **do**
    if $(r_T \notin \mathcal{R}_{dc}$ **and** $\bar{r_T} \notin \mathcal{R})$ **then**
        Let $[u, v]$ be the path of T corresponding to the internal branch of r_T;
        Mark all branches of the path $[u, v]$;
Remove from T branches that have been marked above;
return T**;**

Algorithm 2: Procedure ensuring that the tree T does not contain any branch contradicting triplets in the set $\mathcal{R}$.

CIC (T, n)

$nr_{T,n} \leftarrow 1$;
Let I the set of internal nodes of T;
foreach $u \in I$ **do**
    $c \leftarrow |children(u)|$;
    for j *in* $[2, c]$ **do**
        $nr_{T,n} \leftarrow (nr_{T,n} * (2 * j - 3))$;
$max \leftarrow n - |L(T)|$; $j \leftarrow |L(T)|$;
for k *in* $[1, max]$ **do**
    $nr_{T,n} \leftarrow (nr_{T,n} * (2 * j - 1))$;
    $j \leftarrow j + 1$;
$nr_n \leftarrow (2n - 1)!!$
return $-\log(nr_{T,n}/nr_n)$

Algorithm 3: Procedure computing the CIC value of a tree T, when source tree taxa contain n leaves.

```
betterCIC(T, n, R, R_dc, u, t, above)
if above then
    T' ← T with t inserted above u;
else
    T' ← T with t inserted on u;
T' ← Check_PC (T', R, R_dc);
T' ← Check_PI (T', R);
if CIC(T', n) > CIC(T, n) then
    return true;
else
    return false;
```

Algorithm 4: Procedure returning true if inserting a taxon t in a tree T leads to a tree T' with a greater CIC value, while satisfying PC and PI (the $\texttt{Check}_{\texttt{PC}}$ and $\texttt{Check}_{\texttt{PI}}$ subroutines ensure it).

```
roundIns(T, 𝒯, R, R_dc, t, all, summary)
change ← false ; n ← |L(𝒯)| ;
foreach u ∈ nodes(T) do
    suppAbv(u) ← 0; suppOn(u) ← 0;
𝒯' ← {T_j ∈ 𝒯 such that t ∈ L(T_j) and L(T_j) ∩ L(T) > 2};
foreach T_j ∈ 𝒯' do
    support(T_j, T, t);
nbMaxAbv ← 0; nbMaxOn ← 0 ;
suppMax ← max_{u∈nodes(T)} ( max(suppAbv(u), suppOn(u)) );
if (suppMax = |𝒯'| or all = false) then
    foreach u ∈ nodes(T) do
        if (suppAbv(u) = suppMax) then nbMaxAbv ++; u_abv ← u ;
        if (suppOn(u) = suppMax)  then nbMaxOn ++;  u_on ← u ;
    if (nbMaxAbv = 1 and nbMaxOn = 0) then
        if (all = true) or (betterCIC(T, n, R, R_dc, u_abv, t, true)) then
            T ← T with t inserted above node u_abv;
            change ← true;
    else if (nbMaxAbv = 0 and nbMaxOn = 1) then
        if (all = true) or (betterCIC(T, n, R, R_dc, u_on, t, false)) then
            T ← T with t inserted on node u_on;
            change ← true;
    else if (nbMaxOn = 1 and nbMaxAbv > 0 and summary = true) then
        AbvMax ← {u ∈ nodes(T) such that suppAbv(u) = suppMax};
        if AbvMax ⊆ Children(u_on) ∪ {u_on} then
            if (all = true) or (betterCIC(T, n, R, R_dc, u_on, t, false)) then
                T ← T with t inserted on u_on;
                change ← true;
if (change and suppMax < |𝒯'|) then
    T ← Check_PC (T, R, R_dc)
return change;
```

Algorithm 5: Details of the `roundIns` procedure. This function tries to insert a given taxa t in the backbone tree T. The insertion is performed only if the source trees containing t all indicate the same zone to graft t and the insertion does not decrease the CIC of the built supertree.

$\texttt{insertion}(T, \mathcal{T}, \mathcal{R}, \mathcal{R}_{dc}, priorityList, all, summary)$
$i \leftarrow 1$;
while $i \leq size(priorityList)$ **do**
    Let t be the ith element in $priorityList$;
    if $roundIns(T, \mathcal{T}, \mathcal{R}, t, all, summary)$ **then**
        remove t from $priorityList$; $i \leftarrow 1$
    $i \leftarrow i + 1$;
$T \leftarrow \texttt{Check}_{\texttt{PC}}$ $(T, \mathcal{R}, \mathcal{R}_{dc})$; $T \leftarrow \texttt{Check}_{\texttt{PI}}$ $(T, \mathcal{R})$;

Algorithm 6: Details of the `insertion` procedure. Taxa not yet inserted in the backbone tree are considered in decreasing priority order. Each time a taxon can be inserted (which is decided by the *roundIns* procedure), the taxa with higher priority (that are not yet inserted) are reconsidered. $\texttt{Check}_{\texttt{PC}}$ and $\texttt{Check}_{\texttt{PI}}$ ensure that the output tree still satisfies PI and PC properties.

$\texttt{PhySIC\_IST}(\mathcal{T})$
$\mathcal{R} \leftarrow rt(\mathcal{T})$;
Let $\mathcal{R}_{dc}$ be the set of triplets $r : r, \bar{r} \in \mathcal{R}$;
$priorityList \leftarrow orderList(L(\mathcal{T}), \mathcal{R})$;
Remove the first two leaves, called a and b, from $priorityList$;
Let T be the rooted tree composed of a root node connected to two leaves a and b;
insertion$(T, \mathcal{T}, \mathcal{R}, \mathcal{R}_{dc}, priorityList, true, false)$;
insertion$(T, \mathcal{T}, \mathcal{R}, \mathcal{R}_{dc}, priorityList, true, true)$;
insertion$(T, \mathcal{T}, \mathcal{R}, \mathcal{R}_{dc}, priorityList, false, false)$;
insertion$(T, \mathcal{T}, \mathcal{R}, \mathcal{R}_{dc}, priorityList, false, true)$;

Algorithm 7: Details of the $\texttt{PhySIC\_IST}(\mathcal{T})$ algorithm. After computing $\mathcal{R}$, $\mathcal{R}_{dc}$, the priority list and the starting backbone tree T, the insertions of taxa are done in four successive steps. These four steps differ on whether a maximum or maximal support is required to insert a taxon (first boolean parameter of the *insertion* algorithm) and whether insertions can temporarily contradict some source trees (second boolean parameter of the *insertion* algorithm).

Complexity of PhySIC_IST.

Denoting by k the number of source trees and by n the number of taxa within the tree collection, the time complexity of *PhySIC_IST* is shown to be $O(n^3(k + n^3))$, *i.e.* the method runs in polynomial time. To prove this statement, the complexity of each *PhySIC_IST* subroutine is detailed.

Complexity of support(T_i, T, t) — Alg. 1

T and T_i have size $O(n)$, hence $L(T)$ and $L(T_i)$ can be obtained in $O(n)$. The least common ancestor (lca) of all pairs of nodes in T can be computed in $O(n)$ (see [1, 2]), then each lca query costs $O(1)$. Other steps involved in this subroutine correspond to a constant number of traversals of parts of the trees T and T_i, each time involving $O(1)$ operations per node and branch. As a result, the complexity of the procedure is $O(n)$.

Complexity of $\texttt{Check}_{\texttt{PC}}(T, \mathcal{R}, \mathcal{R}_{dc})$ *— Alg. 2*

This procedure collapses some branches of the tree T, until T does not directly contradict $\mathcal{R} = rt(\mathcal{T})$, hence $\mathcal{T}$. From lemma 1 in [3], this means that the outputted tree T satisfies PC for $\mathcal{T}$. The set $\mathcal{R}_T$ contains $O(n^3)$ triplets. Checking if r_T, resp. $\bar{r_T}$ is in $\mathcal{R}_{dc}$, resp. in $\mathcal{R}$, or not and obtaining the nodes u, v in T corresponding to r_T can be done in constant time, through lca queries (once the tree has been preprocessed in $O(n)$ [1,2]). Marking branches of the path $[u, v]$ is done in $O(n)$ time, proportionally to the number of branches in that path. This happens at worst for each triplet, hence costs $O(n^4)$ globally. This is then the complexity of the procedure (removing all marked branches of T only requires a single search of T, *i.e.* $O(n)$).

Complexity of $\texttt{CIC}(T, n)$ *— Alg. 3*

This procedure requires $O(n)$ time. In the first outer loop, a number of multiplications equal to the number of branches in the tree are performed (thus requiring $O(n)$ time). The second outer loop performs a multiplication per missing taxa (requiring $O(n)$ time again). Then computing $n_R(n)$ by the traditional formula in phylogenetics to count the number of rooted trees having n leaves is done in $O(n)$ multiplications.

Complexity of $\texttt{betterCIC}(T, n, \mathcal{R}, \mathcal{R}_{dc}, u, t, above)$ *— Alg. 4*

Building T' requires copying T and inserting a taxon t above/on the node u, thus costing $O(n)$ time. The complexity of this subroutine is therefore that of the $\texttt{Check}_{\texttt{PI}}$ and $\texttt{Check}_{\texttt{PC}}$ procedures, *i.e.* $O(n^4)$ (see [3, Thm 2] and above, respectively).

Complexity of $\texttt{roundIns}(T, \mathcal{T}, \mathcal{R}, \mathcal{R}_{dc}, t, all, summary)$ *— Alg. 5*

The function `support` is called for each tree containing the taxon t. In the worst case, *i.e.* t is present in all source trees, this step requires $O(kn)$ time. Among the other step of `roundIns`, the most time consuming operations are `betterCIC` and $\texttt{Check}_{\texttt{PC}}$, which both cost $O(n^4)$ time. So, the total cost of `roundIns` is $O(nk + n^4)$.

Complexity of $\texttt{insertion}(T, \mathcal{T}, \mathcal{R}, \mathcal{R}_{dc}, priorityList, all, summary)$*— Alg. 6*

Each of the n taxa is considered at most $O(n)$ times: a first time and if not inserted, each time another taxa is inserted (as long it is not itself inserted). Overall $O(n^2)$ calls to *roundIns* can be issued, each

costing $O(n(k+n^3))$ time. Thus, the `insertion` procedure runs in $O(n^3(k+n^3))$ time.

Complexity of `PhySIC_IST`$(\mathcal{T})$*— Alg. 7*

In the procedure `PhySIC_IST` the first step consists in computing $\mathcal{R}$ and $\mathcal{R}_{dc}$. This step requires $O(kn^3)$ because each of the k trees in the collection can host $O(n^3)$ triplets. Then, for each taxon t of the collection, $|\mathcal{R}(t)|$ and $|\mathcal{R}_{dc}(t)|$ are computed (see section *Outline of the algorithm*) and the taxa are order in decreasing priority order. The amortized complexity of this task is $O(n^3)$ time. The total cost of `PhySIC_IST` is dominated by the complexity of the `insertion` procedure (that is called a constant number of times) and is therefore $O(n^3(k+n^3))$.

References

1. Harel D, Tarjan RE: **Fast algorithms for finding nearest common ancestors**. *SIAM J. Comput.* 1984, **13**(2):338–355.
2. Bender MA, Farach-Colton M: **The LCA Problem Revisited**. In *LATIN '00: Proceedings of the 4th Latin American Symposium on Theoretical Informatics*, London, UK: Springer-Verlag 2000:88–94.
3. Ranwez V, Berry V, Criscuolo A, Fabre P, Guillemot S, Scornavacca C, Douzery E: **PhySIC: a Veto Supertree Method with Desirable Properties**. *Syst. Biol.* 2007, **56**(5):798–817.
